# Parental sports support and emotional-behavioral problems in preschoolers: the chain mediating roles of exercise habits and parent–child relationships

**DOI:** 10.3389/fpsyg.2026.1768932

**Published:** 2026-03-30

**Authors:** Sicheng Min, Jiahao Dong, Dan Li, Xianxiong Li

**Affiliations:** 1 School of Physical Education, Hunan Normal University, Changsha, China; 2 Physical Education Teaching and Research Group, Changsha Foreign Language School, Changsha, China

**Keywords:** emotional-behavioral problems, exercise habits, parental sports support, parent–child relationship, preschool children

## Abstract

**Background:**

Experiencing emotional-behavioral problems during the preschool years can significantly hinder children's development and learning. While physical activity (PA) is recognized as beneficial for mitigating emotional-behavioral problems, the specific role of parental sports support and its underlying mechanisms remains underexplored. This study examined the relationship between parental sports support and emotional-behavioral problems in preschool children, investigating the individual and chain mediating roles of children's exercise habits and the parent–child relationship.

**Methods:**

A cross-sectional survey was conducted with 4,358 parents of children aged 3–6 years in Hunan Province, China, using convenience sampling (2,252 boys, 2,106 girls; mean age 4.4 years). Variables were measured using the Parental Sports Support Scale, the Preschoolers' Exercise Habits Scale, the Parent–Child Relationship Scale, and the Emotional-Behavioral Problems Scale. A chain mediation model was tested using the PROCESS macro in SPSS.

**Results:**

Parental sports support was significantly and negatively associated with preschoolers' emotional-behavioral problems (β = −0.085, *p* < 0.001). Both children's exercise habits and the parent–child relationship served as partial mediators in this association, with the mediating effect of the parent–child relationship (effect = −0.032, 95% *CI*: [ −0.042, −0.021]) being stronger than that of exercise habits (effect = −0.010, 95% *CI*: [−0.013, −0.007]). Furthermore, a significant chain mediation was identified: parental sports support was linked to fewer emotional-behavioral problems through a sequential pathway involving increased exercise habits, which in turn fostered a more positive parent–child relationship (effect = −0.014, 95% *CI*: [−0.017, −0.011]). The total indirect effect accounted for 65.12% of the total effect.

**Conclusions:**

The findings indicate that parental sports support is associated with lower levels of emotional-behavioral problems both directly and indirectly through its relationship with healthier exercise habits and improved parent–child relationships. These results identify pathways through which parental sports involvement is associated with child mental health and suggest that family-centered physical activity interventions may be a valuable approach for addressing emotional-behavioral problems in early childhood. However, due to the cross-sectional design, this study can identify associations but not causal relationships.

## 1 Introduction

The preschool period constitutes a critical window for psychological development. During this time, children are highly susceptible to various internal and external factors. This vulnerability increases the risk of emotional-behavioral problems such as depression, anxiety, and attention-deficit/hyperactivity disorder (Schaan et al., 2019). Experiencing emotional-behavioral problems in early childhood can adversely affect academic performance, social adjustment, peer relationships, family dynamics, and parent–child bonds. These negative outcomes often persist into adulthood (Arslan et al., 2021; Luby et al., 2009). Notably, the reported prevalence of emotional-behavioral problems among preschool children is substantial and rising globally. Estimates range from 5.7% to 31.64% in various international and Chinese studies (Liu et al., 2021; Sahril et al., 2021). This underscores the urgent need for effective early intervention. Timely identification and remediation of contributing factors can yield significant benefits at a relatively low cost (Egger and Angold, 2006; Kariuki et al., 2017).

Within this context, family-based interventions are paramount. Parental sports support is defined as parents' active facilitation, encouragement, and involvement in their child's physical activities (Hosokawa et al., 2023). This construct is multidimensional, encompassing three interrelated components: behavioral modeling (parents demonstrating active lifestyles through their own exercise habits), logistical facilitation (providing resources, access to facilities, and opportunities for activity), and emotional encouragement (expressing warmth, affirmation, and autonomy support during sports-related interactions) (Bandura, 2004; Sheridan et al., 2014). This integrated focus on both behavioral provision and emotional quality distinguishes parental sports support from narrower concepts such as logistical support alone, which lacks the affective component essential for child motivation, or passive modeling, which lacks active engagement. For preschoolers, participating in physical exercise is inherently dependent on caregivers. The Positive Behavior Support framework posits that behavioral problems often stem from unmet needs. These issues can be addressed by cultivating a supportive family environment (Nylén et al., 2021). Concurrently, substantial evidence highlights regular physical activity (PA) as an effective means of ameliorating emotional-behavioral problems in young children (Song et al., 2016). As a proactive and engaged parenting practice, parental sports support emerges as a crucial positive parenting practice (Sanders et al., 2024). Parental sports support not only meets children's exercise needs but also represents a promising factor associated with fewer emotional-behavioral problems.

The mechanisms linking parental sports support to child emotional-behavioral problems may operate through key intermediary factors. Both insufficient PA and poor parent–child relationships are established risk factors for preschool emotional-behavioral problems (Hinkley et al., 2014; Marinelli et al., 2014; Wenjie Shan et al., 2019). Theoretical frameworks offer explanatory pathways. Self-Determination Theory suggests that supporting a child's basic psychological needs for autonomy, competence, and relatedness fosters positive motivation and well-being (Ryan and Deci, 2000; Stanley et al., 2021). Parental sports support, by providing autonomy-supportive strategies, can promote the internalization of good exercise habits, yielding psychological benefits (Phipps et al., 2025b; Vega-Díaz et al., 2023). Complementarily, Attachment Theory emphasizes that secure parent–child bonds are forged through positive communication and shared interactions (Ainsworth and Bowlby, 1991). Joint physical activities facilitated by parental support may enhance this bond, which is in turn associated with lower emotional risk (Davidson et al., 2024; Lee et al., 2025).

Despite this conceptual grounding, significant research gaps remain. While studies on familial correlates of preschool emotional-behavioral problems in China have examined factors like socioeconomic status, family structure, and general parenting styles (Ling et al., 2023; Wang L. et al., 2024), the specific role of parental sports support and its underlying mechanisms is underexplored. Furthermore, empirical research on the impact of family physical education on child mental health is scarce. Although Positive Behavior Support is applied in schools, its adaptation within family contexts, particularly through physical activity, is less studied (Nylén et al., 2021).

Therefore, this study aims to address these gaps by investigating the pathways through which parental sports support is associated with emotional-behavioral problems in preschool children. Specifically, we posit that preschoolers' exercise habits and the parent–child relationship serve as critical mediating variables in this association. By examining both their individual and sequential (chain) mediating roles, this research seeks to elucidate the pathways of influence. The findings are expected to enhance parental understanding of family-based physical education and inform the development of targeted family sports intervention programs for preventing and reducing emotional-behavioral problems in early childhood.

### 1.1 Parental sports support and preschoolers' emotional-behavioral problems

Theoretical and empirical evidence suggest a direct link between family support and child mental health. According to the main effects model of social support network theory, familial support confers direct, positive benefits on children's psychological well-being; enhancing such support consistently leads to improved mental health outcomes (Warheit, 1979). Within this framework, parental sports support is conceptualized as a specific, active form of family support that integrates multiple mechanisms. Unlike general parental involvement, which may be diffuse or passive, parental sports support entails deliberate behaviors aimed at directly influencing children's physical activity engagement (Liu et al., 2017). It encompasses a range of concrete behaviors, including modeling exercise habits, personally supervising or co-participating in activities, purchasing sports-related materials, assisting in developing fitness plans, engaging in sports-related communication, and providing financial resources for sports venues or training. Importantly, what distinguishes parental sports support from related concepts such as physical activity socialization (a broader cultural transmission process) or logistical support (a unidimensional resource provision) is its simultaneous attention to behavioral enactment and emotional quality—the “what” and the “how” of parental involvement in the sports domain (Bandura, 2004; Sheridan et al., 2014; Hosokawa et al., 2023).

Parental sports support thus integrates verbal encouragement, behavioral modeling, and tangible resource provision within family physical education, collectively signifying a positive and affirming parental attitude toward the child's sports participation. As a proactive and engaged parenting approach, it aligns with and nurtures the innate playful disposition of preschool children, contrasting with negative familial approaches such as hostility, neglect, or indifference (Vega-Díaz et al., 2023; Mårs et al., 2024). Prior research corroborates the beneficial role of such support, indicating that parental sports role-modeling, co-participation, and financial facilitation are positively associated with children's mental health (Beets et al., 2010; Yang et al., 2025). Based on the theoretical premise of the main effects model and supporting empirical evidence, we propose that parental sports support is negatively associated with emotional-behavioral problems in early childhood. Consequently, this study proposes Hypothesis 1: Parental sports support will significantly and negatively predict emotional-behavioral problems in preschool children.

### 1.2 The mediating role of preschoolers' exercise habits

Establishing healthy exercise habits early in life is fundamental for holistic child development. National guidelines, such as *The Exercise Guidelines for Preschool Children (3–6 years old)*, recommend substantial daily physical activity, outdoor time, limited screen exposure, and adequate sleep to foster well-being. Adherence to such guidelines reflects good exercise habits, which are associated with lower levels of anxiety and depression and contribute positively to self-concept and emotion regulation (Hirooka et al., 2021). Empirical evidence consistently links preschoolers' lifestyles with their mental health, identifying excessive screen time and insufficient sleep as risk factors (Xiang et al., 2022; Zhou S. et al., 2024), while increased outdoor activity and reduced sedentary behavior are linked to better social skills and fewer emotional-behavioral problems (Rodriguez-Ayllon et al., 2019; Hinkley et al., 2018). Therefore, cultivating good exercise habits forms an essential foundation for preschoolers' physical and mental health.

The formation of these habits in young children is profoundly influenced by parental input. Self-Determination Theory posits that an individual's perception of support from significant others predicts behavioral adherence (Ryan et al., 2009), and interventions based on autonomy support effectively promote health habits (Wang Y. et al., 2024). In the context of physical activity, the development of exercise habits in preschoolers is therefore highly dependent on parental sports support—a construct that subsumes but extends beyond mere modeling. While parents' own exercise habits can predict children's habits through observational learning (Laukkanen et al., 2018; Garriguet et al., 2017), active parental support—through verbal encouragement, shared participation, and logistical facilitation—has been shown to have an even more significant impact (Trost et al., 2003; Gustafson and Rhodes, 2006).

This influence operates through complementary mechanisms. Observational learning theory suggests that children imitate parental behaviors, meaning parents act as direct sports role models (Qunito Romani, 2020). Furthermore, children perceive and value supportive parental behaviors such as companionship and praise. When parents provide autonomy-supportive sports environments that satisfy children's psychological needs, it enhances the child's intrinsic motivation and creates positive experiences associated with physical activity (Van Petegem et al., 2015).

In summary, parental sports support—manifested through role modeling, co-participation, communication, and resource provision—helps meet children's emotional needs in physical activity, stimulates their interest and motivation, and directly promotes the formation of good exercise habits. These habits, in turn, ensure adequate levels of beneficial physical activity while displacing risk factors like excessive screen time and sedentariness, ultimately linked to fewer emotional-behavioral problems. Based on this rationale, this study proposes Hypothesis 2: Preschool children's exercise habits mediate the relationship between parental sports support and preschool children's emotional-behavioral problems.

### 1.3 The mediating role of the parent–child relationship

Empirical evidence robustly identifies the parent–child relationship as a key predictor of emotional-behavioral problems in early childhood. For instance, studies have shown that higher-quality parent–child interactions are associated with fewer behavioral and peer-relationship problems and more prosocial behaviors (Poulain et al., 2019). Large-scale prospective research indicates that difficulties in the parent–child relationship constitute an independent risk factor for later psychiatric diagnoses (Holstein et al., 2021), and longitudinal data confirm that poor parent–child interaction in early years predicts subsequent emotional-behavioral problems (Schimmenti and Bifulco, 2015).

Given its foundational role, fostering a positive parent–child relationship is a central focus in developmental research. Shared family activities, particularly physical activities, have been shown to enhance parent–child intimacy and strengthen relational bonds (Dorsch et al., 2017; Knoester and Randolph, 2019; Lisinskiene and Lochbaum, 2019). Parental sports support, characterized by shared physical engagement and positive emotional exchange, provides a structured context for such beneficial interaction, thereby promoting attachment security and relationship quality (Ling et al., 2023). Conversely, parental behaviors that detract from engagement, such as excessive phone use, can diminish emotional communication and hinder relationship development (Liu et al., 2024). The relational context is so pivotal that family closeness has been identified as a mediator in the intergenerational transmission of physical activity patterns (Pan, 2022).

In summary, the parent–child relationship functions as a primary emotional conduit between parent and child. The physical co-engagement and positive affect inherent in parental sports support are posited to enhance this relationship. An improved parent–child relationship, in turn, is associated with a more secure and supportive environment, which relates to fewer emotional-behavioral problems. Therefore, this study proposes Hypothesis 3: The parent–child relationship plays a mediating role between parental sports support influencing preschool children's emotional-behavioral problems.

### 1.4 The chain mediating role of preschoolers' exercise habits and parent–child relationships

As established, both preschoolers' exercise habits and the quality of the parent–child relationship are significant factors in the development of emotional-behavioral problems and serve as mediating pathways through which parental sports support exerts its influence. However, an important, less explored question is whether these two mediators are sequentially linked. Specifically, does the cultivation of good exercise habits in children subsequently enhance the parent–child relationship? Furthermore, do exercise habits and the parent–child relationship function in tandem as a chain of mediators between parental sports support and child emotional-behavioral problems? Investigating this potential sequential mechanism is a central aim of the present study.

Attachment theory provides a relevant framework, positing that early attachment patterns profoundly affect later socioemotional development (Bowlby, 1982). Secure attachment is associated with fewer mental health difficulties, while insecure attachment predicts greater internalizing and externalizing problems (Scott et al., 2011; Pace and Zappulla, 2011). Secure attachment is not a static trait but a dynamic construct, actively built and maintained through positive, reciprocal interactions between parent and child.

We propose that parental sports support and child exercise habits interact to foster such positive interactions. Parental sports support primarily represents a top-down process where parents' attitudes and behaviors aim to shape the child's activities. In contrast, a child's established exercise habit can initiate a bottom-up process, where the child's own interest and routine in physical activity creates opportunities and incentives for parental engagement (Li et al., 2025). When a child, influenced by initial parental support, develops a sustained exercise habit, this habit can, in turn, lead the child to seek further shared activity and support from the parent. This increased mutual engagement—stemming from both the parents' supportive stance and the child's active habit—necessitates and fosters more frequent contact, cooperative communication, and shared positive experiences (Knoester and Fields, 2020; Li et al., 2024). Such reciprocal interactions are the bedrock of secure attachment, thereby contributing to a more stable, intimate, and higher-quality parent–child relationship (Li et al., 2025). This enhanced relationship then constitutes a more proximal protective factor against emotional-behavioral problems.

In summary, preschoolers' exercise habits can be viewed as a successful outcome of parental sports support. More importantly, these habits create a feedback loop that deepens the parent–child bond. The intersection of parental provision (support) and child initiation (habit) builds a closer, more interactive relationship, which itself is a key determinant of emotional and behavioral health. Therefore, this study proposes Hypothesis 4: Preschool children's exercise habits and parent–child relationships play a chain-mediated role in the association between parental sports support and preschool children's emotional-behavioral problems.

### 1.5 The current study

**Figure 1:**
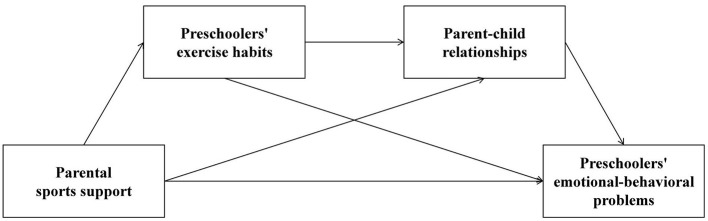
The hypothesis chain mediation model.

To summarize, the present study was designed to investigate the pathways linking parental sports support to emotional-behavioral problems in preschool children. Situated within the frameworks of self-determination theory and attachment theory, we propose a chain mediation model (see Figure 1) in which parental sports support is associated with fewer emotional-behavioral problems through two sequential mechanisms: first by fostering children's exercise habits, and subsequently by enhancing the quality of the parent–child relationship. This model integrates the direct pathway and the mediating roles of exercise habits and parent–child relationships—both independently and in sequence—into a unified conceptual framework, allowing for a nuanced understanding of how parental involvement in physical activity relates to early childhood mental health.

## 2 Method

### 2.1 Participants and procedure

The cross-sectional survey was conducted in November–December 2023. Using convenience sampling, 21 public kindergartens were selected across seven prefecture-level cities in Hunan Province: Changsha, Zhuzhou, Xiangtan, Changde, Hengyang, Yiyang, and Yueyang. Questionnaires were completed by the fathers or mothers of children enrolled in the surveyed kindergartens. The questionnaire was administered online to parents of young children with the informed consent of the kindergarten director, the head teacher, the children, and their parents. All procedures involving human participants in this study were approved by the Ethics Committee of Hunan Normal University (approval number: 2023 – 386).

During the official test, the kindergarten organized a parents' meeting with the parents of the children in the class under investigation. The kindergarten teacher in charge of the class was responsible for introducing the guidelines to the parents in detail, explaining the purpose of the questionnaire and the norms for completing it, and demonstrating the filling in of the example questions to make sure that the parents filled in the questions truthfully. At this meeting, parents were also informed that participation was entirely voluntary, that they could withdraw at any time without consequence, and that all data would be kept confidential and used solely for research purposes. Written informed consent was obtained from each parent prior to questionnaire administration. After explaining the situation, parents scanned the QR code or the link to the questionnaire and filled it out on the spot. The main teacher monitored the quality of the questionnaire during the administration process, and the questionnaire completion time was limited to about 15 min.

A total of 4,926 questionnaires were recovered and 568 invalid questionnaires such as inconsistent answers to trap questions, apparently random filling, and answering time of less than 6 min were excluded, resulting in 4,358 valid questionnaires, which is a valid questionnaire rate of 88.5 per cent. The specific situation of the test families is shown in Table 1.

**Table 1:** Characteristics of demographic variables in the tested families.

Variables	*M* (*SD*) or *N* (%)
Age (years)	4.4 ± 1.004
3	984 (22.6%)
4	1,377 (31.6%)
5	1,303 (29.9%)
6	692 (15.9%)
Sex	
Boy	2,252 (51.6%)
Girl	2,106 (48.4%)
Residence	
Urban	2,163 (49.6%)
Rural	2,195 (50.4%)
Family structure	
Nuclear family	2,360 (54.1%)
Extended family	1,888 (43.3%)
Reconstituted family	38 (0.9%)
Single-parent family	72 (1.7%)
Parents' education level	
Junior high school and below	256 (5.9%)
Middle or high school	651 (14.9%)
Associate degree	1,119 (25.7%)
Bachelor's degree	2,058 (47.2%)
Master's degree and above	274 (6.4%)
Monthly household income level	
Low-income level	129 (2.9%)
Lower-middle-income level	730 (16.8%)
Middle-income level	975 (22.4%)
Middle-upper-income level	1,780 (40.8%)
Upper-income level	744 (17.1%)

Basic information of survey respondents (*N* = 4,358).

*M*, mean; *SD*, standard deviation; *N*, absolute frequency; %, relative frequency.

### 2.2 Measures

#### 2.2.1 Parental sports support

We used the “Youth Sunshine Physical Activity Long-term Mechanism Model-Family Sports and Health Education Scale” developed by Zheng et al. (2015), which contains three dimensions: “parental sports awareness”, “parental sports behavior”, and “family sports economy”. Referring to the classification in Li Jiawei's paper (Li and Liang, 2020), the two dimensions of (e.g., “I am in the habit of exercising”) and “Family Sports Economy” (e.g., “I support my child exercising in a paid venue”) were combined to form the “Parental Sports Support” scale. The scale consists of a total of 13 items and is scored on a 5-point Likert scale, where 1 = very non-compliant and 5 = very compliant, with higher scores indicating better parental sport support behaviors. The internal consistency reliability of the Parental Sports Support Scale in this study was 0.908 Cronbach's alpha.

#### 2.2.2 Preschoolers' exercise habits

We used the “planned exercise habits” dimension from the “Early Childhood Physical Lifestyle” scale developed by Lv et al. (2020) to measure “preschool children's exercise habits”. The scale has a total of 4 items, for example, “The number of times in a week my child engages in habitual physical activity”, “Duration of my child's habitual physical activity to date”, “The duration of my child's habitual physical activity on a single occasion”, “The cumulative number of hours per week that my child performs habitual physical activity”. Notify parents in advance that habitual sports activities refer to any activities that are intentionally pre-arranged in daily life, rather than casual, temporary, and simple activities. A 5-point Likert scale was used, with 1–5 representing different numbers of times and durations according to the different questions in the questionnaire, with higher scores indicating a higher level of exercise habit formation in preschoolers. The Cronbach's alpha for internal consistency of the children's exercise habits scale in this study was 0.782.

#### 2.2.3 Parent–child relationships

We used the Parent–Child Relationship Scale, which was developed by Pianta et al. (1997) and validated by Zhang et al. (2023). The scale totals 26 items and contains two dimensions of parent–child closeness (e.g., “My child and I have a close and affectionate relationship”) and parent–child conflict (e.g., “My child and I always seem to be at each other's throats”). A 5-point Likert scale was used, where 1 = very poorly met and 5 = very well met. In the present study, the scores of the parent–child conflict dimension were reverse-scored and added to the parent–child intimacy dimension to obtain the total parent–child relationship score, based on the scoring method of Zu et al. (2022). A higher total score indicates a better parent–child relationship. The internal consistency reliability Cronbach α of the parent–child relationship scale in this study was 0.849.

#### 2.2.4 Emotional-behavioral problems

We selected the first four difficulty factors from the Strengths and Difficulties Questionnaire, developed by Goodman (1997), to form the “Emotional Behavioral Problems” scale. There are a total of 20 question items, including emotional symptoms (e.g., “The child has a lot of worries and often shows apprehension”), conduct problems (e.g., “The child has frequent tantrums or fussiness”), hyperactive attention inability (e.g., “The child is restless, overactive, and unable to remain quiet for long periods.”), and peer interaction problems (e.g., “The child is quite lonely and often plays by himself”). Each entry is rated on a three-point scale of 0, 1, and 2, where 0 = does not meet, 1 = somewhat meets, and 2 = fully meets. Higher scores indicate more severe emotional-behavioral problems in preschoolers. The Cronbach's alpha for internal consistency of the Emotional Behavior Problems Scale in this study was 0.779.

#### 2.2.5 Basic family information

This part is mainly information on demographic variables, including preschool children's gender, urban and rural factors, family structure, monthly family income, parents' education level, and parents' occupation. The type of family structure, urban–rural factor, and socio-economic status of the family have been confirmed to have a significant effect on the main study variables (Wang et al., 2016), therefore, the type of family structure, urban–rural factor, and socio-economic status of the family have been used as control variables in this study. Among them, the level of monthly family income, parent's education level, and occupation constitute the family's socio-economic status. Referring to the scoring model of scholar (Zhou N. et al., 2024), the three types of scores are first standardized and then summed up to finally get the indicator of family's socio-economic status.

### 2.3 Data analysis

Through SPSS 25.0 software, the data from 4,358 valid questionnaires recovered were entered and statistically analyzed. (1) internal consistency coefficient test, descriptive analysis of data, and reliability and validity test of the scale were conducted. (2) A common method bias test was conducted by Harman one-way method to prevent potential bias problems during data collection. (3) Pearson correlation analysis was used to test the correlations among parental sports support, preschool children's exercise habits, parent–child relationships, preschool children's emotional behavioral problems, and other control variables. (4) The direct effect of parental sports support on preschool children's emotional-behavioral problems was assessed by regression analyses, and the chained mediation effect test was conducted using Model 6 in the PROCESS macro program developed by Hayes, and the significance level of the mediation effect was tested using the Bootstrap method.

## 3 Results

### 3.1 Common method deviation test

As the data in this paper were obtained through parental self-report and the results may be affected by common method bias, the data were tested for common method bias using Harman's one-factor method. Principal component analysis was used to conduct exploratory factor analysis of the full question load of parental sports support, preschool children's exercise habits, parent–child relationship, and emotional-behavioral problems. The results of the analysis showed that 11 factors with an eigenroot greater than 1 were extracted, and the first factor explained 15.11% of the variance, which was less than the critical point of 40%, indicating that there was no common method bias problem in this study.

### 3.2 Descriptive statistics and correlation analysis

The prerequisite for exploring the mediating effect is the existence of a correlation between the variables, and the results of Pearson's correlation coefficient between the variables are shown in Table 2. There is a significant positive correlation between parental sports support, preschoolers' exercise habits, and parent–child relationship (*P* < 0.001), and a significant negative correlation between preschool children's emotional behavioral problems and parental sports support, preschoolers' exercise habits, and parent–child relationship (*P* < 0.001).

**Table 2:** Descriptive statistics and correlations of study variables.

Variables	1	2	3	4
1. Parental sports support	0.908	–	–	–
2. Preschoolers' exercise habits	0.249^***^	0.782	–	–
3. Parent–child relationships	0.156^***^	0.244^***^	0.849	–
4. Preschoolers' emotional-behavioral problems	−0.169^***^	−0.238^***^	−0.599^***^	0.779
*M*	45.30	13.10	100.14	11.00
*SD*	9.316	3.602	11.990	5.207

*N* = 4,358, ^***^P < 0.001, ^**^P < 0.01, ^*^P < 0.05, the diagonal represents the Cronbach's alpha.

### 3.3 The chain mediation model

Chained mediation effect tests were conducted using Model 6 in the PROCESS program in SPSS. The results, as shown in Table 3, showed that parental sports support significantly negatively predicted preschoolers' emotional behavioral problems (β = −0.085, *P* < 0.001) as well as significantly positively predicted preschoolers' exercise habits (β = 0.089, *P* < 0.001) in Model 1 and Model 2. In Model 3, both parental sports support (β = 0.129, *P* < 0.001) and preschoolers' exercise habits (β = 0.629, *P* < 0.001) significantly and positively predicted the parent–child relationship. In Model 4, parental sports support (β = −0.030, *P* < 0.001), preschoolers' exercise habits (β = −0.113, *P* < 0.001), and parent–child relationship (β = −0.246, *P* < 0.001) all significantly negatively predicted emotional-behavioral problems in preschoolers.

**Table 3:** Test for the chain mediation model.

Model	Regression equation		Overall fit indices			Significance		
	Outcome variables	Predictive variables	*R*	*R^2^*	*F*	β	*t*	95% *CI*
1	Preschoolers' emotional-behavioral problems	Parental sports support	0.227	0.052	59.397	−0.085	−10.287^***^	[−0.102, −0.069]
2	Preschoolers' exercise habits	Parental sports support	0.338	0.114	140.538	0.089	15.966^***^	[0.078,0.100]
3	Parent–child relationships	Parental sports support	0.288	0.083	78.955	0.129	6.668^***^	[0.091,0.167]
		Preschoolers' exercise habits				0.629	12.246^***^	[0.528,0.729]
4	Preschoolers' emotional-behavioral problems	Parental sports support	0.614	0.377	439.329	−0.030	−4.298^***^	[−0.044, −0.016]
		Preschoolers' exercise habits				−0.113	−6.028^***^	[−0.149, −0.076]
		Parent-child relationships				−0.246	−45.413^***^	[−0.257, −0.236]

*N* = 4,358; ^***^P < 0.001, Regression coefficients and significance of control variables are omitted for space limitations.

**Figure 2:**
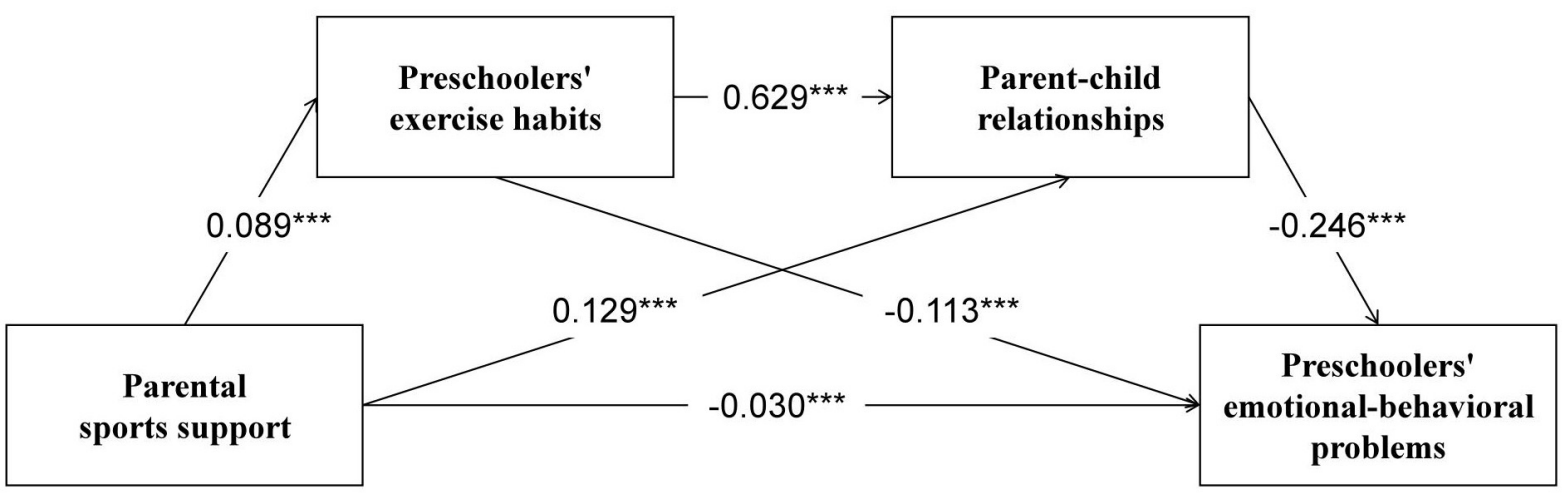
The moderated chain mediation model. *N* = 4,358, ***P < 0.001.

The Bootstrap method was used to repeat the extraction 5,000 times to calculate 95% confidence intervals respectively, and the mediation effect was established if the 95% confidence interval of the indirect effect did not contain zero. The results are shown in Table 4. The mediated effect size of preschoolers' exercise habits was −0.010 (*95% CI*: [−0.013, −0.007]), accounting for 11.63% of the total effect size. The mediating effect size for the parent–child relationship was −0.032 (*95% CI*: [−0.042, −0.021]), accounting for 37.21% of the total effect size. The chain-mediated effect size for exercise habits and parent–child relationship for preschoolers was −0.014 (*95% CI*: [−0.017, −0.011]), accounting for 16.28% of the total effect size. Since the 95 % confidence intervals of all mediation effects did not include zero, the chain mediation of exercise habits and parent–child relationships in preschoolers was statistically significant. Examination of effect sizes revealed that the mediating effect of the parent–child relationship (effect = −0.032) was approximately three times larger than that of exercise habits alone (effect = −0.010), and the chain mediation effect (−0.014) fell between these two. The total indirect effect accounted for 65.12% of the total effect. The chain mediation model diagram is shown in Figure 2.

**Table 4:** The direct and indirect effect of chain mediation model.

Effect types	Intermediary path	Effect	Boot SE	Relative mediation effect	95% *CI*
Total effect		−0.086	0.008	100%	[−0.102, −0.069]
Direct effect		−0.030	0.007	34.88%	[−0.044, −0.016]
Total indirect effect		−0.056	0.006	65.12%	[−0.066, −0.044]
Mediating effect 1	PSS → PEH → PEBP	−0.010	0.002	11.63%	[−0.013, −0.007]
Mediating effect 2	PSS → PCR → PEBP	−0.032	0.005	37.21%	[−0.042, −0.021]
Mediating effect 3	PSS → PEH → PCR → PEBP	−0.014	0.001	16.28%	[−0.017, −0.011]

PSS, parental sports support; PEH, preschoolers' exercise habits; PCR, parent–child relationships; PEBP, preschoolers' emotional-behavioral problems.

## 4 Discussion

### 4.1 The negative and significant predictors of parental sports support on preschoolers' emotional-behavioral problems

The results supported Hypothesis 1, Parental sports support was negatively associated with preschoolers' emotional-behavioral problems, consistent with theoretical frameworks on parental involvement. This form of support—encompassing behaviors like co-participation, supervision, and resource provision—constitutes positive, active parental engagement. According to parental involvement theory, such engagement reflects parental commitment and is integral to children's psychological development (El Nokali et al., 2010; Grolnick and Slowiaczek, 1994). It can be posited that this supportive involvement is associated with lower risks associated with insufficient parenting or non-optimal parenting styles, which are known contributors to emotional-behavioral problems (Guo et al., 2022; Yang and Tahir, 2023).

Empirical evidence corroborates the benefits of this form of involvement. General parental autonomy support and active engagement have been linked to improved cognitive abilities, are linked to lower levels of depression and anxiety, fewer behavioral problems, and enhanced self-esteem and prosocial behaviors in young children (Vasquez et al., 2016; Daniel et al., 2016; Laucht et al., 1997). Conversely, disruptions in supportive parenting are associated with negative emotional and cognitive states in children, undermining their emotional regulation (Markoulaki et al., 2025). Specific to physical activity, intervention studies indicate that parent–involved family games can ameliorate emotional-behavioral problems (Schneider et al., 2022), and parent–child sports activities have shown positive effects on improving attachment behaviors (Chen, 2025).

In essence, parental sports support may function as a concrete manifestation of positive parental involvement and autonomy support. It conveys care and affirmation to the child, potentially enhancing their sense of security and providing a constructive context for healthy socioemotional development. This may partly explain its observed association with fewer emotional-behavioral problems.

### 4.2 The negative mediation effect of preschoolers' exercise habits

The analysis supported Hypothesis 2, preschoolers' exercise habits emerged as one pathway through which parental sports support is associated with fewer emotional-behavioral problems. This finding aligns with research demonstrating the benefits of regular physical activity for mental health (Wang, 2022; Zou et al., 2024).

First, the established benefits of regular physical activity for mental health provide a foundation for understanding this association. Research confirms that consistent activity is effective in mitigating emotional-behavioral problems across developmental stages (Biddle et al., 2019). The mechanisms are multifaceted. Psychologically, it can be linked to lower anxiety and stress, which are associated with higher self-efficacy and self-esteem, aid in attention regulation, and facilitate positive peer interactions (Guzmán-Muoz et al., 2025; White et al., 2024). Physiologically, it may be related to better emotional and behavioral regulation by enhancing cortical stimulation and cerebral blood flow (Tari et al., 2020), modulating neuroendocrine systems (Chen and Nakagawa, 2023), and bolstering executive functions (Gapin and Etnier, 2014). Consequently, children with higher levels of physical activity typically exhibit fewer behavioral issues and greater psychological resources than their less active peers (Rodriguez-Ayllon et al., 2019).

Second, Self-Determination Theory explains how parental support translates into sustained child habits. Parental sports support represents a direct response to the child's activity needs, creating an autonomy-supportive environment that satisfies core psychological needs and fosters the internalization of motivation (Rodrigo et al., 2024). This supportive context is crucial for habit formation, a finding echoed in related research showing that parental support often outweighs child motivation in predicting activity levels (Phipps et al., 2025a; Liu et al., 2023), and that parental autonomy support positively influences adolescents' physical activity behavior and adherence (Dong and Mao, 2018). For preschoolers specifically, whose primary social world is the family, parental influence on habit formation is particularly potent (Lv et al., 2020; Wouter et al., 2011).

In summary, parental sports support is associated with the development of good exercise habits by providing modeling, opportunity, and motivational support. These established habits, in turn, ensure regular engagement in physical activity, which operates through concurrent psychological and physiological mechanisms to are linked to lower risk of emotional-behavioral problems. It is also worth considering alternative interpretations. Children who already exhibit fewer emotional-behavioral problems may be more receptive to developing exercise habits, and parents may find it easier to provide support to such children. This bidirectional possibility cannot be ruled out, given the cross-sectional design. Future longitudinal research could help clarify the direction of these relationships.

### 4.3 The negative mediation effect of parent–child relationships

The results supported Hypothesis 3, parent–child relationship emerged as the strongest mediating pathway linking parental sports support to emotional-behavioral problems. This finding aligns with the spillover hypothesis from family systems theory, which posits that dynamics within one family subsystem can transfer to and affect other subsystems (Erel and Burman, 1995). In this context, supportive sports behaviors from parents likely “spill over” into the parent–child subsystem, enriching that relationship, which subsequently influences the child's individual outcomes (Pu and Rodriguez, 2021).

The pathway can be understood in two sequential parts. First, parental sports support is associated with better parent–child relationships. Such support, characterized by co-participation, encouragement, and shared positive affect, helps meet children's basic psychological needs, particularly for autonomy and relatedness (Vasquez et al., 2016). When children perceive their parents as supportive allies in physical activity, it is linked to greater intimacy and less conflict (Smith and Cté, 2024), a pattern consistent with the benefits of authoritative and interest-based parenting styles (Wang, 2023). Second, the quality of the parent–child relationship itself is a well-established predictor of child adjustment. A positive, secure relationship provides a foundation of safety and support, facilitating healthy socialization and emotional regulation. Conversely, a poor relationship is a recognized risk factor for emotional-behavioral problems, as it can lead children to perceive their environment as threatening or chaotic, increasing vulnerability to behavioral and emotional difficulties (Jiang et al., 2025).

Rohner's acceptance-rejection theory offers a parsimonious explanation for this mediating role (Khaleque and Rohner, 2002). Under conditions of high parental sports support, children are more likely to interpret parental involvement as a sign of acceptance and care, promoting the internalization of social norms and reducing problematic behaviors. When parental support is low, the same interactions may be perceived as intrusive or rejecting, fostering negative cognitive schemas that elevate the risk for emotional-behavioral problems.

In summary, parental sports support promotes a closer, more positive parent–child relationship. This enhanced relationship creates a familial environment conducive to healthy socioemotional development, thereby may function as a pathway through which parental support is associated with fewer emotional-behavioral problems in preschool children. However, an alternative explanation warrants consideration. Families with higher-quality relationships may simply be more inclined to engage in shared physical activities, rather than the activities themselves driving relationship quality. In this view, a positive parent–child relationship serves as a precondition for, rather than an outcome of, parental sports support. The cross-sectional nature of this study cannot distinguish between these possibilities, and longitudinal research is needed to establish temporal precedence.

### 4.4 The negative chain mediation effect of preschoolers' exercise habits and parent–child relationships

The analysis confirmed Hypothesis 4, a sequential pathway was also identified, in which parental sports support was associated with children's exercise habits, which in turn related to parent–child relationship quality, ultimately linking to fewer emotional-behavioral problems. This finding extends understanding of how these factors may operate together. Notably, this finding underscores that the parent–child relationship serves as a proximal mediator, receiving positive influence from both initial parental support and the child's subsequent behavioral habit.

Bowlby's attachment theory provides a coherent framework for understanding this sequential mechanism (Bowlby, 1982). Secure parent–child attachment, a lasting emotional bond cultivated through positive interactions, is foundational for healthy socioemotional development (Groh et al., 2017). Children with secure attachments derive satisfaction and security from their family relationships, which fosters positive social adaptation and reduces emotionally problematic behaviors (Kerns and Brumariu, 2014). The formation of this attachment is heavily dependent on the pattern of parent–child interactions (Wu et al., 2022).

The identified chain mediation reflects the construction of such a positive interaction pattern. Parental sports support represents a parent–initiated, top-down investment in the child's activity. When this support is effective, it helps the child internalize good exercise habits. These child-initiated habits then create a bottom-up dynamic: the child's routine and interest in physical activity naturally invite further parental engagement, communication, and shared experience. This reciprocity—where parental provision meets child initiation—fosters the frequent, positive, and cooperative interactions essential for building a robust and secure attachment. Importantly, children are active agents in this process; they employ strategies to maintain closeness with caregivers (Bowlby, 1982). In this context, a well-established exercise habit can be viewed as a proactive behavioral strategy through which the child seeks and sustains rewarding interactions with the parent.

These findings suggest that parental sports support may contribute to a pattern of positive parent–child interaction through its association with children's exercise habits. This pattern, in turn, is linked to better emotional and behavioral outcomes. However, the cross-sectional design means that alternative sequences—such as better-behaved children eliciting more parental support and shared activity—cannot be ruled out.

### 4.5 Implications and limitations

This study offers several practical implications for families seeking to support preschool children's mental health through sports involvement, along with directions for future research. First, the findings underscore that parental sports support is a feasible and low-risk strategy for promoting emotional well-being in early childhood. Rather than relying on resource-intensive interventions, families can adopt simple, consistent supportive behaviors—such as regularly engaging in physical activities with the child, offering verbal encouragement, and ensuring access to safe play environments—to be associated with fewer emotional-behavioral problems. Critically, the effectiveness of these behaviors depends on whether children perceive them as expressions of genuine parental warmth and care. Therefore, interventions and family education programs should emphasize not just what parents do, but how they do it—encouraging a warm, responsive, and autonomy-supportive style during sports-related interactions. Second, the findings suggest that fostering children's exercise habits requires more than instruction or logistical support. Parents are encouraged to be attuned to their child's emotional cues and interests during physical activity. By prioritizing quality companionship and open communication, parents can transform sports participation into a shared, enjoyable experience. This not only promotes habit formation but also strengthens the parent–child bond—an essential developmental asset in its own right. Finally, given the strong mediating role of the parent–child relationship, interventions aimed at reducing emotional-behavioral problems should consider the relationship itself as a primary target. Shared sports activities offer a natural and effective context for enhancing relational quality. Programs designed to support parents could include practical guidance on how to use physical activity as a platform for building trust, reducing conflict, and fostering emotional closeness. By focusing on both the child's behavioral routines and the emotional quality of parent–child interactions, family-based sports interventions may yield more sustainable benefits for early childhood mental health.

Several limitations of this study should be acknowledged. First, all data were derived from parent-reported questionnaires, which may introduce potential common method bias. In this large-scale survey, it was impractical to assess the study variables through observation, experiments, or interviews. However, these constructs are inherently family-context specific, making parents the most knowledgeable informants regarding their children's behaviors and experiences. Second, the study did not differentiate between maternal and paternal roles. Given that fathers and mothers may influence children's sports participation and parent–child attachment in distinct ways, future research should examine these potential differences. Third, the cross-sectional design precludes definitive causal inferences. Longitudinal studies are needed to verify the directionality of the proposed relationships and to explore the long-term effects of parental sports support, child exercise habits, and parent–child relationship quality on developmental outcomes. Fourth, this study did not examine whether demographic variables—such as children's age, gender, or family socioeconomic status—moderate the proposed mediation pathways, as these factors may influence the strength of the observed relationships. However, it is worth noting that all participants were recruited from public kindergartens across several cities within the same province in China, which may have reduced variability in educational environments, regional policies, and cultural backgrounds. This relative homogeneity in the sample context may have helped to partially mitigate the potential confounding effects of these demographic factors. It should also be noted that the convenience sampling approach and geographic restriction to Hunan Province may limit representativeness, and parenting practices related to children's sports involvement may vary across different socio-cultural contexts.

## 5 Conclusion

This study identifies multiple pathways through which parental sports support is associated with fewer emotional-behavioral problems in preschool children. A direct association was observed, and indirect associations were found through children's exercise habits and parent–child relationship quality, with the latter showing a stronger mediating effect. Furthermore, a sequential pathway was identified: parental support was associated with children's exercise habits, which in turn were linked to parent–child relationship quality, and this sequence was related to fewer emotional-behavioral problems. These findings highlight parental involvement in physical activity as a potentially valuable component of family-based strategies for supporting early childhood mental health.

## Data Availability

The raw data supporting the conclusions of this article will be made available by the authors, without undue reservation.
